# The Accuracy of Emergency Physicians’ Suspicions of Active Pulmonary Tuberculosis

**DOI:** 10.3390/jcm10040860

**Published:** 2021-02-19

**Authors:** Shiang-Jin Chen, Chun-Yu Lin, Tzu-Ling Huang, Ying-Chi Hsu, Kuan-Ting Liu

**Affiliations:** 1 Department of Emergency Medicine, Kaohsiung Medical University Hospital, Kaohsiung Medical University, Kaohsiung 80708, Taiwan; paperdietsharon@gmail.com; 2 Division of Infectious Diseases, Department of Internal Medicine, Kaohsiung Medical University Hospital, Kaohsiung Medical University, Kaohsiung 80708, Taiwan; infectionman@gmail.com; 3 School of Medicine, College of Medicine, Kaohsiung Medical University, Kaohsiung 80708, Taiwan; 4 Infection Control Center, Kaohsiung Medical University Hospital, Kaohsiung Medical University, Kaohsiung 80708, Taiwan; 1030276@kmuh.org.tw (T.-L.H.); 1030082@kmuh.org.tw (Y.-C.H.); 5 Center for Tropical Medicine and Infectious Diseases Research, Kaohsiung Medical University, Kaohsiung 80708, Taiwan

**Keywords:** pulmonary tuberculosis, emergency department, recognition, delayed isolation

## Abstract

Objective: To investigate factors associated with recognition and delayed isolation of pulmonary tuberculosis (PTB). Background: Precise identification of PTB in the emergency department (ED) remains challenging. Methods: Retrospectively reviewed PTB suspects admitted via the ED were divided into three groups based on the acid-fast bacilli culture report and whether they were isolated initially in the ED or general ward. Factors related to recognition and delayed isolation were statistically compared. Results: Only 24.94% (100/401) of PTB suspects were truly active PTB and 33.77% (51/151) of active PTB were unrecognized in the ED. Weight loss (*p* = 0.022), absence of dyspnea (*p* = 0.021), and left upper lobe field (*p* = 0.024) lesions on chest radiographs were related to truly active PTB. Malignancy (*p* = 0.015), chronic kidney disease (*p* = 0.047), absence of a history of PTB (*p* = 0.013), and lack of right upper lung (*p ≤* 0.001) and left upper lung (*p* = 0.020) lesions were associated with PTB being missed in the ED. Conclusions: Weight loss, absence of dyspnea, and left upper lobe field lesions on chest radiographs were related to truly active PTB. Malignancy, chronic kidney disease, absence of a history of PTB, and absence of right and/or left upper lung lesions on chest radiography were associated with isolation delay.

## 1. Introduction

Tuberculosis (TB) remains a widespread infectious disease and global public health issue. According to a World Health Organization (WHO) report published in 2020, an estimated 10.0 million people had TB in 2019, equivalent to 130 cases (range, 116–143) per 100,000 people [1] Compared with countries that have a high TB burden, which have 150–400 cases per 100,000 population, Taiwan has an intermediate TB burden with an incidence of 48.4 new cases per 100,000 population in 2014. Furthermore, approximately one-fourth of the global population is infected with *Mycobacterium tuberculosis* [1].

Patients with active pulmonary TB may make multiple emergency department (ED) visits [2]. In previous studies, a high proportion (39%) of patients presented to the ED prior to their diagnosis [3] and TB was presumed in 71% of patients admitted from the ED [4]. Moreover, patients not being admitted to the ED represent a risk factor for prolonging isolation delay [5]. These studies emphasize that physicians in the ED play the role of gatekeepers in performing the first-line detection of patients with TB. Delayed recognition and isolation in the ED will lead to disease transmission between patients and healthcare workers.

The main objectives of this study were to determine the number and proportion of cases of TB suspected by emergency physicians of truly being TB and the number of missed diagnoses of TB by ED physicians. Furthermore, we identified and described characteristics associated with either recognition or delayed isolation of pulmonary TB (PTB) in the ED.

## 2. Materials and Methods

### 2.1. Study Setting

We conducted a retrospective study including 1077 consecutive non-trauma adults (age ≥ 18 years) admitted to the isolation ward between 1 January 2015, and 31 December 2017. We aimed to study factors associated with recognition and delayed isolation of patients with active PTB who were initially admitted from the ED. Our exclusion criteria were (i) admission from the outpatient department (OPD), (ii) patients admitted to the intensive care unit (ICU), (iii) patients with human immunodeficiency virus (HIV), (iv) patients referred from other hospitals due to suspected or diagnosed PTB, and (v) patients with active TB under treatment. This single-center study was conducted at a tertiary referral hospital center and university-affiliated teaching hospital located in southern Taiwan. There were 47,244 non-trauma patients admitted to hospital via ED during the study period. There were two negative pressure isolation rooms in the ED and 15 negative pressure isolation rooms in the isolation ward. Ethical approval and a waiver of the need for informed consent were obtained from the Institutional Review Board (serial number: KMUHIRB-E(I)-20190335) of the hospital.

### 2.2. Definition of the Study Population

The emergency physicians suspected patients had infectious TB based on clinical histories and chest X-ray findings initially. Then, the patients were put in an isolation area and admitted to the isolation ward. Acid-fast smear and TB cultures were performed in an isolation area or isolation ward. The TB might have been excluded after three sets of sputum examination over 3 days. As the patients usually stayed in ED only for a few hours, these TB examination results came out after admission. Patients who were not suspected of having TB would not receive a TB examination in the ED.

We enrolled one group of patients who were suspected of having infectious TB by an emergency physician and another group of patients who were confirmed to have TB after hospital admission but not suspected to have TB in the ED. We focused on the population of non-critical and non-HIV patients with a new or recurrent episode of TB in the ED.

In our study, patients suspected of having PTB by an emergency physician and AFB (acid-fast bacilli) sputum smears, culture tests, and preemptive airborne isolation were initiated in the ED before admission to isolation wards and classified in the “TB suspect group,” which was further divided into two subgroups: “TB isolated group,” where the sputum culture was positive for *M. tuberculosis*, and the “non-TB isolated group” Further intergroup comparisons between the TB isolated and non-TB isolated patients were performed. 

In patients who were not suspected as having TB while in the ED but were subsequently suspected of having TB after hospital admission, AFB sputum smears, culture tests, and immediate transfer to the airborne isolation ward were performed in the general ward. Patients with a culture-confirmed diagnosis of TB were included in the “delayed isolation group.” Investigating differences between the TB isolated group and delayed isolation group might help identify factors associated with delayed isolation. 

### 2.3. Data Collection and Definitions 

We collected data from the electronic medical records of patients admitted to the isolation ward from the ED and general ward during the selected period. A diagnosis of pulmonary TB was based on a sputum culture of respiratory specimens positive for *M. tuberculosis*. The median time from ED triage to the initiation of isolation ward admission was recorded.

We focused on potential predictors associated with TB by evaluating factors including demographics (age, sex), comorbidities (smoking habit, diabetes mellitus, end-stage renal disease, lung cancer, malignancy, chronic kidney disease, and history of PTB), initial presenting symptoms (cough, expectoration, dyspnea, fever, chillness, hemoptysis, weight loss, chest pain, night sweats, and no symptoms), chest X-ray (CXR) results categorized in terms of the involved field (right upper, right middle, right lower, left upper, and left lower lung fields), cavities on chest radiography, performance of chest computed tomography (CT) in the ED, and laboratory findings (leukocytosis, elevated C-reactive protein [CRP], anemia).

“No symptom” is defined as having a lack of symptoms or symptoms not being perceived as being TB-specific. In our study, patients with a history of PTB were defined as those with a previous diagnosis of TB who had completed treatment or were declared cured [6]. Newly diagnosed PTB was defined as: (i) patients had never been treated or diagnosed with TB and (ii) patients with a history of PTB diagnosed with a recurrent episode during hospitalization [6]. Patients with active pulmonary TB were defined as those who had documented infectious TB and had not completed anti-tuberculosis treatment.

Recognition of TB was mostly based on CXRs, which were interpreted by a resident emergency physician or attending emergency physician and were used to determine the need for isolation. We collected CXR results recorded in the radiology report. A normal pattern was defined as the absence of any abnormal lesion on CXR. Typical chest X-rays were defined as infiltrates or consolidations and/or cavities in the right and/or left upper lungs. Atypical findings were defined as radiological manifestations other than upper lung involvement.

### 2.4. Statistical Analysis

Categorical variables are expressed as numbers (percentage), and continuous variables as means ± standard deviation (SD) or medians with interquartile range (IQR). Univariate comparisons were performed using the Chi-square test or Fisher’s exact test for categorical variables and independent samples *t*-test for continuous variables. The associated factors were first assessed using univariate analysis. Factors with *p* < 0.05 were included in the multivariable logistic regression model. Odds ratios (ORs) and 95% confidence intervals (CIs) were calculated. All tests of significance were two-sided, and *p* < 0.05 was considered statistically significant. All analyses were performed using MedCalc Statistical Software version 19.4.1 (MedCalc Software Ltd., Ostend, Belgium). 

## 3. Results

**Figure 1:**
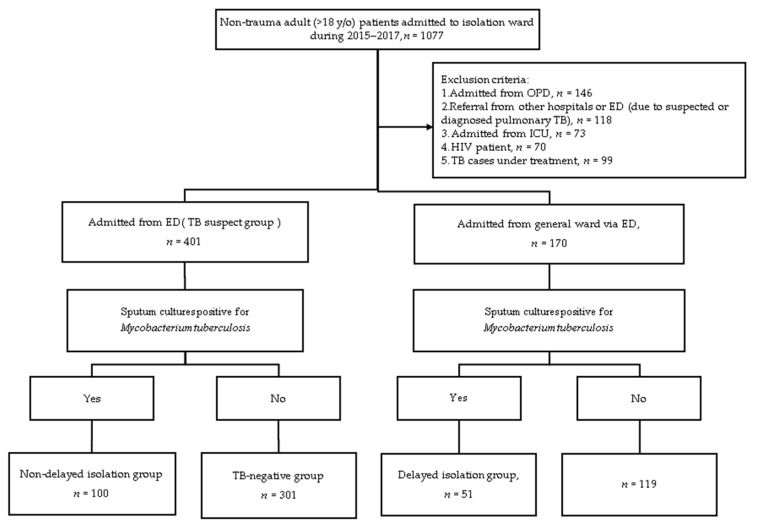
Flow chart of case enrollment.

After applying the exclusion criteria shown in Figure 1, 401 patients were admitted to isolation wards via ED, and 170 patients were admitted to isolation wards from the general ward. Of the 170 patients transferred to the isolation ward during hospitalization, 51 (30%) patients had positive sputum cultures. In the delayed isolation group, the mean interval between presentation to the ED and initiation of isolation ward admission was 6 days (interquartile range (IQR): 5–14 days).

### 3.1. Factors Associated with the Recognition of Pulmonary Tuberculosis (PTB) in the Emergency Department (ED)

Table 1 shows the baseline patient characteristics of the TB suspect groups and the comparison between the TB isolated and non-TB isolated groups. Of the 401 patients suspected of having PTB by ED physicians, 100 (24.94%) had sputum cultures positive for *M. tuberculosis*, and 301 (75.06%) had negative reports. The median age of patients was 66 years (mean age, 66.24 ± 17.89) and age was independently associated with the recognition of TB. Approximately 71% (286/401) of the patients were men and men (*p* = 0.027) accounted for a significantly higher proportion of the TB isolated group (80%, 80/100). Regarding comorbidities, we found that nearly half of the TB suspect group had a smoking habit (47.9%; 192/401) and one-fourth had a history of TB (21.9%; 88/401). However, there were no statistical differences between the TB and non-TB isolated groups in terms of comorbidities. Although approximately three-fourths of patients in the TB suspect group had a cough (73.8%; 296/401), patients in the TB isolated group had significantly fewer respiratory complaints of cough (*p* = 0.04) and dyspnea (*p* = 0.003) than patients in the non-TB isolated group. Approximately one-fifth (20.9%; 84/401) of patients in the TB suspect group had weight loss, and weight loss was more frequently noted in the TB isolated group (*p* = 0.023) than in the non-TB isolated group.

Left upper lung (*p* = 0.014) field predominance was identified as a chest radiographic finding that was useful for detecting active PTB. In contrast, cavitary (*p* = 0.837) lesions on CXR showed no association with PTB. Approximately 29.4% (118/401) of patients in the TB suspect group underwent chest CT examination, and the chest CT scan results were not significantly different between the TB isolated and non-TB isolated groups. Regarding laboratory data, leukocytosis was a significant factor associated with being in the non-TB isolated group (*p* = 0.039).

In the multivariate analysis shown in Table 2, patients who had weight loss (adjusted odds ratio [OR] = 1.91, 95% confidence interval [CI]: 1.10–3.31, *p* = 0.021) and left upper lung (adjusted OR = 1.71, 95% CI: 1.07–2.75, *p* = 0.027) involvement on CXR were more likely to have PTB. However, patients without PTB were significantly more likely to present with dyspnea (adjusted OR = 0.53, 95% CI: 0.30–0.91, *p* = 0.021) than those in the TB isolated group. 

### 3.2. Factors Associated with Delayed Isolation of PTB in the ED

Table 3 shows intergroup comparisons of demographic and clinical characteristics in the TB isolated group and delayed isolation group. On univariate analysis, patients with delayed isolation were older than those in TB isolated group (mean age, 76.4 ± 14.6 vs. 66.0 ± 17.9 years; *p* < 0.001). Therefore, renal function impairment (*p* = 0.02) was more likely observed in the delayed isolation group. Most patients (78.8%; 119/151) were men, but sex distribution was not statistically significantly different between the two groups. Regarding comorbidities, patients with a history of cancer (*p* = 0.017) had a significantly higher risk of delayed isolation, while those with a history of PTB (*p* = 0.022) were less likely to have delayed isolation.

The most common symptom of PTB was a cough (58.9%; 89/151), which was more prevalent in the TB-isolated group than in the delayed isolation group (66% vs. 45%; *p* = 0.013). Moreover, patients in the TB isolated group were significantly more likely to have a clinical history of weight loss than those in the delayed isolation group (29% vs. 7%; *p* = 0.037). Of the 151 TB-positive patients, 20 (13.2%) had no symptoms. Interestingly, asymptomatic presentation was not a risk factor for delayed isolation.

PTB was diagnosed on the basis of chest radiography. In the retrospective review of chest radiographs, PTB was not detected early in the initial CXR in approximately one-third of patients (51/151; 33.75%). Typical presentations such as right upper lung field (*p* < 0.001) involvement, left upper lung field (*p* = 0.001) involvement, and cavitation (*p* = 0.019) were more likely to be directly admitted to the isolation room in the ED than in the delayed isolation group. Among atypical findings on CXR, the right middle lung (*p* = 0.041) and right lower lung (*p* < 0.001) were more frequently noted in the delayed isolation group. 

In the multiple logistic regression model in Table 4, a history of cancer (adjusted OR = 6.09, 95% CI: 1.63–22.77, *p* = 0.007) and CKD (adjusted OR = 4.69, 95% CI: 1.02–21.44, *p* = 0.047) were factors significantly associated with an increased risk of isolation delay. In contrast, patients with a history of PTB infection (adjusted OR = 0.11, 95% CI: 0.03–0.46, *p* = 0.002), and right upper lung field (adjusted OR = 0.16, 95% CI: 0.05–0.41, *p* = 0.001) and left upper lung field (adjusted OR = 2.98, 95% CI: 1.19–7.46, *p* = 0.02) involvement on CXR had a lower risk of delayed recognition of TB in the ED.

### 3.3. Analysis of Delayed Isolation Subgroup

We noticed that each patient in the delayed isolation group underwent chest radiography while in the ED; therefore, we further explored each radiographic finding. Among the 51 patients, 32 (63%) had atypical findings, 12 (24%) had pure right upper lobe and/or left upper lobe lesions, and seven (13%) had normal CXRs. Of the seven patients with normal CXR, two had no symptoms while in the ED and developed symptoms related to upper respiratory tract infection during hospitalization. Notably, 12 patients with typical CXR findings of upper lobe infiltrates, which predict a diagnosis of PTB, were not detected by emergency physicians. 

Our results suggest that of all patients with new or recurrent PTB (N = 151), 66.23% (N = 100) of them were identified by emergency physicians and isolated immediately in the ED. These data suggest that a large proportion of patients with infectious TB were admitted from the ED.

## 4. Discussion

Emergency physicians suspecting TB were found to have a sensitivity of 66.75% and positive predictive value of 24.94%. As there were 47,244 non-trauma patients admitted to hospital via ED during the study period, the specificity and negative predictive value would be more than 99%. The results showed that weight loss, absence of dyspnea, and left upper lobe field lesions on chest radiographs can contribute to the recognition of active PTB. In contrast, malignancy, CKD, absence of history of PTB, and absence of right and/or left upper lung lesions were likely to lead to diagnostic delay. Despite numerous studies on delayed isolation of PTB, our study was innovative in evaluating the effect of the same factors on recognition and delayed isolation simultaneously. Moreover, we excluded ICU and HIV patients, a group of patients in whom CXR findings are particularly atypical [7,8], to evaluate CXR interpretation for PTB by ED physicians.

In the TB suspect group, weight loss was a significant factor associated with the recognition of PTB, which is consistent with previous studies [2,9,10]. Malnutrition causes reduced proliferation of T-cells and impaired cell-mediated immunity [11], which in turn leads to increased susceptibility to infection. In a population-based survey of TB in Los Angeles County, 44.5% of patients had weight loss and 40.6% had anorexia [10]. In accordance with our findings, a study in Denmark showed that weight loss alerts the physician to initiate a careful evaluation of suspected TB, which was associated with a short health system delay (<1 week) [12].

Among the TB suspect group, our results showed that only pathology in the left upper lung field on CXR was significantly related to the detection of PTB; however, a number of studies suggest that the right upper lung field, left upper lung field, and cavitary lesions on chest radiographs are independently associated with an increased risk of AFB-positive smear findings [9,13,14]. However, in our study, the presence of lung cavities on CXR had no association with the recognition of active TB. The lack of this association could be because not all cavitation was related to TB disease. In one recent study using high-resolution CT (HRCT) for predicting positive-culture PTB, lower lobe cavitary lung lesion, except S6, was a negative predictor for PTB [15]. Furthermore, this study demonstrated that non-cavitation findings, such as consolidation in S1, S2, S1+S2 and S6 and clusters of nodules/masses in S1, S2, S1+2, had the strongest association with culture-positive TB [15]. 

A history of malignancy was found to be associated with delayed isolation, which is consistent with the results from previous population-based observations in Korea [16,17]. In our delayed isolation group, approximately one-fourth (21.6%, 10/51) of the patients were diagnosed with malignancy. The reason for the low rate of suspicion was probably because the underlying malignancy will divert the attention of ED physicians from clinical features related to PTB. In our study, cancer was not a significant predictor of TB. However, a recent study highlights the increased risk of active TB in all patients with cancer [18]. In the last decade, the incidence of TB in Taiwan has decreased by approximately 40–50%; however, TB infection in people with cancer has increased from 3% in 2000 to 13% in 2015 [19]. Furthermore, the recurrence rate of TB in this population was as high as 5.03% within 2 years in a high TB incidence setting [19]. Therefore, ED physicians should more precisely evaluate patients diagnosed with malignancy and consider TB in the differential diagnosis.

The probability of delayed diagnosis of PTB on CXR was significantly higher among patients without right and/or left upper lung field lesions. Previous studies reported that upper zone lesions on chest radiographs were related to early isolation [17] and had the shortest time to isolation (3.3 days; *p* = 0.015) [13]. In our model, all patients in the delayed isolation group underwent CXR in the ED before admission. We found that 86% (44/51) of the delayed isolation group had an abnormality on a chest radiograph. In addition, 63% (32/51) had an atypical presentation on chest radiographs. As a result, atypical chest radiographs not only caused delayed isolation in the ED but also caused a prolonged delay prior to isolation after admission [5]. Han et al. reported that only 23% of patients with active PTB had typical radiological findings [16]. This result highlights the importance of carefully reviewing all imaging undertaken in the ED and the necessity of routine chest radiograph preadmission, especially in a country with a high TB prevalence [20].

In our study, approximately one-third (51/151; 33.75%) of chest radiographs of PTB were initially interpreted as normal (7/51; 13%) or other diagnoses by emergency physicians. It should be noted that 12 (12/51; 24%) patients had pure upper respiratory tract lesions on chest radiographs in the delayed isolation group. According to a recent study based on a retrospective review of chest radiographs, the accuracy of CXR-based diagnosis of PTB in all patients was 71% (92/130), and PTB was not diagnosed on initial CXR in 29% (38/130) [17]. A chest radiograph with PTB may be inaccurately interpreted by a physician [21]. This result suggests that improving the ability of emergency physicians to interpret chest radiographs will have a great effect on the early detection of PTB.

CT of the thorax was not performed unless a differential diagnosis of TB was suspected. In our study, further chest CT examination had a low predictive value of neither recognition nor delayed diagnosis of PTB. Nam et al. also found that there was no significant relationship between chest CT acquisition and delayed isolation of PTB [17]. Regarding chest CT for TB detection, our findings differ from previous reports that HRCT can promptly diagnose culture-positive pulmonary TB [15,17]. A possible explanation for this difference is that the CT images were interpreted by highly experienced radiologists in these studies. Therefore, consulting radiologists about the chest CT interpretation of PTB before isolation can probably improve the accuracy of PTB isolation.

In this study, the median time until isolation in patients who remained undiagnosed and untreated was 6 days (IQR: 5–14 days). In comparison, previous studies from countries with low TB incidence reported a median in-hospital delay of 33 days [12] and 31 days [22]. The reason for our shorter diagnostic delay is probably due to greater clinical experience with and alertness regarding TB in our region, which has an intermediate TB status. Potential misdiagnosis was more common in hospitals with fewer TB cases [23]. Physicians with less experience recognizing TB may be less likely to make an accurate diagnosis, especially in a time-limited setting, such as the ED. 

There were two strengths of the study. First, our TB cases were defined by positive culture reports instead of AFB smears due to the low sensitivity of direct smear methods. Many TB patients have negative AFB smears with a subsequent positive culture [24]. One recent study in Korea found that in culture-confirmed PTB patients, 37% of patients had negative AFB smear results [16]. Therefore, our study can more accurately demonstrate the factors related to PTB. Second, we focused on non-ICU and non-HIV patients. In the HIV-positive population with acquired immunodeficiency syndrome (AIDS), PTB disease may present with atypical findings or with no lesions on chest radiography [7,25]. On the other hand, ICU patients more often than not have abnormal chest radiograph findings and are at risk of ICU-related complications [8]. Our study can more accurately evaluate ED physicians’ performance in interpreting tuberculosis images.

Despite our important findings, there are several limitations to this study. First, this study was based on a single-center cohort with a small sample size, which limits the generalizability of our findings to other institutions with different prevalence or incidence rates of TB. Second, given the retrospective nature of this study design, the completion and accuracy of documented charts were limited. In addition, information on social risk factors and details regarding previous TB diagnosis and treatment were unavailable. Third, the mean diagnostic delay is probably underestimated because we have excluded critical patients. Those patients tended to have a complex radiological appearance [8,14] and a longer diagnostic delay [14]. Fourth, TB identification needs learning and experience. This study did not put the individual differences between physicians into a variable. However, the rate of delay isolation in our study is not inferior to previous literature [12,22,23].

## 5. Conclusions

In our study, emergency physicians had a sensitivity of 66.75% and positive predictive value of 24.94% of PTB identification. Among patients in the ED suspected of having TB, weight loss, absence of dyspnea, and left upper lobe field lesions on CXR were related to active PTB. Malignancy, CKD, absence of a history of PTB, and absence of right and/or left upper lung lesions on chest radiography were associated with ED missing. Clinical suspicion of PTB requires comprehensive history taking and precise radiologic diagnosis. However, most of the clinical features and radiological findings of PTB were not specific. Therefore, rapid, accurate, and inexpensive diagnostic tools are necessary to reduce diagnostic delay and control the in-hospital spread of TB.

## Figures and Tables

**Table 1:** Demographic characteristics of the tuberculosis (TB) suspect group in the emergency department (ED), comparing the TB isolated group with the non-TB isolated group.

Characteristics	Total (*n* = 401)	TB Isolated (*n* = 100)	non-TB Isolated (*n* = 301)	*p*-Value
Demographic				
Age ^a^ (years)	66.24 ± 17.89	66.0 ± 17.9	66.3 ± 17.9	0.960 ^c^
Male sex ^b^	286 (71.3)	80 (80.0)	206 (68.4)	0.027 ^c^
Comorbidities				
Smoker ^b^	192 (47.9)	49 (49.0)	143 (47.5)	0.796 ^c^
DM ^b^	143 (35.7)	22 (22.0)	82 (27.3)	0.293 ^c^
CKD ^b^	46 (11.5)	7 (7.0)	39 (13.0)	0.106 ^c^
ESRD ^b^	13 (3.2)	2 (2.0)	11 (3.7)	0.532 ^d^
Lung cancer ^b^	9 (2.2)	2 (2.0)	7 (2.3)	1.000 ^d^
Malignancy ^b^	47 (11.7)	8 (8.0)	39 (13.0)	0.182 ^c^
History of PTB ^b^	88 (21.9)	23 (23.0)	65 (21.6)	0.769 ^c^
Clinical symptoms				
Cough ^b^	296 (73.8)	66 (66.0)	230 (76.4)	0.040 ^c^
Expectoration ^b^	233 (58.1)	50 (50.0)	183 (60.8)	0.058 ^c^
Dyspnea ^b^	146 (36.4)	24 (24.0)	122 (40.5)	0.003 ^c^
Fever ^b^	180 (44.9)	42 (42.0)	138 (45.8)	0.503 ^c^
Chillness ^b^	55 (13.7)	14 (14.0)	41 (13.6)	0.924 ^c^
Hemoptysis ^b^	50 (12.5)	8 (8.0)	42 (14.0)	0.119 ^c^
Weight loss ^b^	84 (20.9)	29 (29.0)	55 (18.3)	0.023 ^c^
Chest pain ^b^	58 (14.5)	19 (19.0)	39 (13.0)	0.137 ^c^
Night sweats ^b^	5 (1.2)	1 (1.0)	4 (1.3)	1.000 ^d^
No symptom ^b^	32 (8.0)	12 (12.0)	20 (6.6)	0.087 ^c^
The involved field on chest X-ray (CXR)				
Right upper field ^b^	300 (74.8)	68 (68.0)	232 (77.1)	0.070 ^c^
Right middle field ^b^	108 (26.9)	25 (25.0)	83 (27.6)	0.616 ^c^
Right lower field ^b^	120 (29.9)	24 (24.0)	96 (31.9)	0.136 ^c^
Left upper field ^b^	194 (48.4)	59 (59.0)	135 (44.9)	0.014 ^c^
Left lower field ^b^	116 (28.9)	31 (31.0)	85 (28.2)	0.598 ^c^
Others				
Cavity on CXR ^b^	38 (9.5)	10 (10.0)	28 (9.3)	0.837 ^c^
Chest CT in ED ^b^	118 (29.4)	26 (26.0)	92 (30.6)	0.386
Laboratory findings				
Leukocytosis ^e^	176 (43.9)	35 (35.0)	141 (46.8)	0.039 ^c^
Elevated CRP ^f^	206 (51.4)	49 (49.0)	157 (52.2)	0.584 ^c^
Anemia ^g^	90 (22.4)	19 (19.0)	71(23.6)	0.341 ^c^

^a^ Data presented as mean ± standard deviation (median, range). SD, standard deviation. ^b^ Data are presented as *n* (%). ^c^ Chi-square test. ^d^ Fisher’s exact test. ^e^ Leukocytosis defined as white blood cell count > 10,000 cells/μL. ^f^ Elevated C-reactive protein (CRP) defined as CRP > 50 mg/dL. ^g^ Anemia defined as hemoglobin < 10 g/dL. TB, tuberculosis; PTB, pulmonary tuberculosis; DM, diabetes mellitus; CKD, chronic kidney disease; ESRD, end-stage renal disease. In our study, the diagnosis of comorbidities were based on the 10th revision of the International Statistical Classification of Diseases. CXR, chest X ray; CT, computed tomography; ED, emergency department.

**Table 2:** Variables associated with recognition of PTB in TB suspect group.

Characteristics	Adjusted OR	95% CI for OR	*p*-Value
Demographic			
Age			
≥65 years vs. ≤65 years	0.85	0.52–1.36	0.472
Sex			
male vs. female	1.70	0.96–3.01	0.069
Clinical symptoms			
Cough			
yes vs. no	0.61	0.36–1.02	0.061
Dyspnea			
yes vs. no	0.52	0.30–0.89	0.017
Weight loss			
yes vs. no	1.89	1.09–3.27	0.023
The involved field on CXR			
Left upper field			
yes vs. no	1.77	1.10–2.86	0.019
Laboratory findings			
Leukocytosis			
yes vs. no	0.65	0.40–1.06	0.083

Logistic regression model including all factors *p* < 0.05 in the univariate analysis in Table 1. CI, confidence interval; OR, odds ratio; CI, confidence interval. Reference group (OR = 1). Leukocytosis defined as white blood cell count > 10,000 cells/μL. CXR, chest X-ray.

**Table 3:** Demographic characteristics of PTB patients in TB isolated group compared with delayed isolation group.

Characteristics	Total (*n* = 151)	TB Isolated (*n* = 100)	Delayed Isolation (*n* = 51)	*p*-Value
Demographic				
Age ^a^ (years)	71 ± 17.6	66.0 ± 17.9	76.4 ± 14.6	<0.001 ^c^
Male sex ^b^	119 (78.8)	80 (80.0)	39 (76.5)	0.616 ^c^
Comorbidities				
Smoker ^b^	66 (43.7)	49 (49.0)	17 (33.3)	0.067 ^c^
DM ^b^	40 (26.5)	22 (22.0)	18 (35.3)	0.081 ^c^
CKD ^b^	17 (11.3)	7 (7.0)	10 (19.6)	0.020 ^c^
ESRD ^b^	5 (3.3)	2 (2.0)	3 (5.9)	0.336 ^d^
Lung cancer ^b^	3 (2.0)	2 (2.0)	1 (2.0)	1.000 ^d^
Malignancy ^b^	19 (12.6)	8 (8.0)	11 (21.6)	0.017 ^c^
History of PTB ^b^	27 (17.9)	23 (23.0)	4 (7.8)	0.025 ^d^
Clinical symptoms				
Cough ^b^	89 (58.9)	66 (66.0)	23 (45.1)	0.013 ^c^
Expectoration ^b^	70 (46.3)	50 (50.0)	20 (39.2)	0.210 ^c^
Dyspnea ^b^	44 (29.1)	24 (24.0)	20 (39.2)	0.052 ^c^
Fever ^b^	69 (45.7)	42 (42.0)	27 (52.9)	0.203 ^c^
Chillness ^b^	22 (14.6)	14 (14.0)	8 (15.7)	0.781 ^c^
Hemoptysis ^b^	11 (7.2)	8 (8.0)	3 (5.9)	0.751 ^d^
Weight loss ^b^	36 (23.8)	29 (29.0)	7 (13.7)	0.037 ^c^
Chest pain ^b^	27 (17.9)	19 (19.0)	8 (15.7)	0.616 ^c^
Night sweats ^b^	1 (0.7)	1 (1.0)	0 (0.0)	1.000 ^d^
No symptom ^b^	20 (13.2)	12 (12.0)	8 (15.7)	0.528 ^c^
Involved field on CXR				
Right upper field ^b^	88 (58.3)	68 (68.0)	20 (39.2)	<0.001 ^c^
Right middle field ^b^	46 (30.5)	25 (25.0)	21 (41.2)	0.041 ^c^
Right lower field ^b^	51 (33.8)	24 (24.0)	27 (52.9)	<0.001 ^c^
Left upper field ^b^	75 (49.7)	59 (59.0)	16 (31.4)	0.001 ^c^
Left lower field ^b^	48 (31.8)	31 (31.0)	17 (33.3)	0.771 ^c^
Others				
Cavity on CXR ^b^	10 (6.6)	10 (10.0)	0 (0.0)	0.017 ^d^
Chest CT in ED ^b^	33 (21.9)	26 (26.0)	7 (13.7)	0.085 ^c^
Laboratory findings				
Leukocytosis ^e^	53 (35.1)	35 (35.0)	18 (35.3)	0.971 ^c^
Elevated CRP ^f^	79 (52.3)	49 (49.0)	30 (58.8)	0.254 ^c^
Anemia ^g^	31 (20.5)	19 (19.0)	12 (23.5)	0.516 ^c^

^a^ Data presented as mean ± standard deviation (median, range). SD, standard deviation. ^b^ Data are presented as n (%). ^c^ Chi-square test. ^d^ Fisher’s exact test. ^e^ Leukocytosis defined as white blood cell count > 10,000 cells/μL. ^f^ Elevated C-reactive protein (CRP) defined as CRP > 50 mg/dL. ^g^ Anemia defined as hemoglobin < 10 g/dL. TB, tuberculosis; PTB, pulmonary tuberculosis. DM, diabetes mellitus; CKD, chronic kidney disease; ESRD, end-stage renal disease. In our study, the classification of comorbidities was based on the 10th revision of the International Statistical Classification of Diseases. CXR, chest X ray; CT, computed tomography; ED, emergency department.

**Table 4:** Factors associated with delayed isolation among patients with culture-positive tuberculosis.

Characteristics	Adjusted OR	95% CI for OR	*p*-Value
Demographics			
Age			
≥65 years vs. ≤65 years	1.84	0.67–5.01	0.236
Sex			
male vs. female	0.93	0.33–2.61	0.897
Comorbidities			
CKD			
yes vs. no	4.68	0.98–22.23	0.053
Malignancy			
yes vs. no	4.49	1.33–15.18	0.016
History of PTB			
yes vs. no	0.18	0.05–0.65	0.009
Clinical symptoms			
Cough			
yes vs. no	0.54	0.22–1.35	0.188
Weight loss			
yes vs. no	0.36	0.12–1.12	0.078
Involved field on CXR			
Right upper field			
yes vs. no	0.19	0.07–0.52	0.001
Right middle field			
yes vs. no	3.13	0.76–12.88	0.11
Right lower field			
yes vs. no	1.70	0.47–6.19	0.421
Left upper field			
yes vs. no	0.35	0.14–0.88	0.025

Logistic regression model including all factors *p* < 0.05 in univariate analysis in Table 4. CI, confidence interval; OR, odds ratio; CI, confidence interval. Reference group (OR = 1). Leukocytosis defined as white blood cell count > 10,000 cells/μL. CXR, chest X-ray.

## Data Availability

The data presented in this study are available on request from the corresponding author.
